# Polymorphism in *INSR* Locus Modifies Risk of Atrial Fibrillation in Patients on Thyroid Hormone Replacement Therapy

**DOI:** 10.3389/fgene.2021.652878

**Published:** 2021-06-23

**Authors:** Enrique Soto-Pedre, Moneeza K. Siddiqui, Cyrielle Maroteau, Adem Y. Dawed, Alex S. Doney, Colin N. A. Palmer, Ewan R. Pearson, Graham P. Leese

**Affiliations:** ^1^Division of Population Health and Genomics, School of Medicine, Ninewells Hospital and Medical School, University of Dundee, Dundee, United Kingdom; ^2^Centre for Pharmacogenetics and Pharmacogenomics, Ninewells Hospital and Medical School, University of Dundee, Dundee, United Kingdom; ^3^Medicines Monitoring Unit and Hypertension Research Centre, Ninewells Hospital and Medical School, University of Dundee, Dundee, United Kingdom; ^4^Department of Endocrinology and Diabetes, Ninewells Hospital and Medical School, University of Dundee, Dundee, United Kingdom

**Keywords:** atrial fibrillation, insulin receptor, thyroid hormone replacement therapy, hypothyroidism, genetics

## Abstract

**Aims:**

Atrial fibrillation (AF) is a risk for patients receiving thyroid hormone replacement therapy. No published work has focused on pharmacogenetics relevant to thyroid dysfunction and AF risk. We aimed to assess the effect of L-thyroxine on AF risk stratified by a variation in a candidate gene.

**Methods and Results:**

A retrospective follow-up study was done among European Caucasian patients from the Genetics of Diabetes Audit and Research in Tayside Scotland cohort (Scotland, United Kingdom). Linked data on biochemistry, prescribing, hospital admissions, demographics, and genetic biobank were used to ascertain patients on L-thyroxine and diagnosis of AF. A GWAS-identified insulin receptor-*INSR* locus (rs4804416) was the candidate gene. Cox survival models and sensitivity analyses by taking competing risk of death into account were used. Replication was performed in additional sample (The Genetics of Scottish Health Research register, GoSHARE), and meta-analyses across the results of the study and replication cohorts were done. We analyzed 962 exposed to L-thyroxine and 5,840 unexposed patients who were rs4804416 genotyped. The rarer G/G genotype was present in 18% of the study population. The total follow-up was up to 20 years, and there was a significant increased AF risk for patients homozygous carriers of the G allele exposed to L-thyroxine (RHR = 2.35, *P* = 1.6e–02). The adjusted increased risk was highest within the first 3 years of exposure (RHR = 9.10, *P* = 8.5e–04). Sensitivity analysis yielded similar results. Effects were replicated in GoSHARE (*n* = 3,190).

**Conclusion:**

Homozygous G/G genotype at the *INSR* locus (rs4804416) is associated with an increased risk of AF in patients on L-thyroxine, independent of serum of free thyroxine and thyroid-stimulating hormone serum concentrations.

## Introduction

Hypothyroidism is the most common thyroid disorder affecting about 3–5% of the general population, and patients are nearly always treated with L-thyroxine (Flynn et al., 2004; Wiersinga et al., 2012). Patients usually respond well to treatment, and dosage is monitored in response to a combination of serum thyroid stimulating hormone (TSH) concentration and patients’ symptoms (Wiersinga et al., 2012). However, increased risk of cardiovascular disease, atrial fibrillation (AF) and bone fractures have been described in patients receiving long-term replacement thyroxine therapy (Flynn et al., 2010; Chaker et al., 2015; Floriani et al., 2017).

Atrial fibrillation is the most common cardiac dysrhythmia and a leading cause of cardiovascular and cerebrovascular morbidity and mortality (Chugh et al., 2014). Risk of AF for patients taking L-thyroxine is partly related to their dose and serum TSH concentration (Flynn et al., 2010). Over the last decade, progress has been made in defining the genetic basis of AF, and there is now evidence that genetic factors may play a role in its pathogenesis. Most published work has focused on identifying genetic variants (common and rare) associated with AF (Fatkin et al., 2017), less on pharmacogenetics relevant to AF management (Huang and Darbar, 2016), few on genetic determinants of thyroid function/dysfunction associated with AF (Ellervik et al., 2019; Larsson et al., 2019; Salem et al., 2019), but none on pharmacogenetics relevant to thyroid dysfunction on AF risk.

We have previously replicated in a Scottish population a number of GWAS-identified loci associated with serum TSH concentrations including the insulin receptor-*INSR* locus (Soto-Pedre et al., 2017). Insulin resistance and serum TSH concentration have both been highlighted as being possible underlying mechanisms for AF (Flynn et al., 2010; Bell and Goncalves, 2019; Bohne et al., 2019; Ellervik et al., 2019; Salem et al., 2019). We aimed to assess the effect of L-thyroxine replacement therapy on AF stratified by a variation at an *INSR* locus.

## Materials and Methods

### Discovery Cohort

A retrospective follow-up study was performed among patients from the Genetics of Diabetes Audit and Research Tayside Scotland (GoDARTS) study. All subjects in this population are of white European ethnicity, the period of follow-up was defined from 1994 to March 2014 and all patients with at least one serum TSH recording were considered for inclusion. For each individual the date of entry into the study was the first date of thyroid replacement therapy prescription (exposed cohort) or the date at first serum TSH recording (unexposed cohort). Each eligible patient was followed from the date of entry until either occurrence of AF or withdrawal from observation (i.e., the earliest of three dates: date of death, last date under observation, or 1 April 2014).

Each patient has a unique identification number (Community Health Index) which facilitates data linkage across all available electronic medical records (EMRs) by the Health Informatics Centre of the University of Dundee^1^. Linked data on biochemistry, prescribing, hospital admissions, demographics, and genetic biobank were used to ascertain patients on L-thyroxine and diagnosis of AF (see Supplementary Material for a detailed description of linked datasets).

### Replication Cohort

The Genetics of Scottish Health Research register (GoSHARE) was used to perform the replication analyses (McKinstry et al., 2017). In brief, participants anywhere in Scotland are asked to allow their information held within NHS Scotland EMRs to be used for research, and to give consent for blood remaining from diagnostic tests to be used. Any participant included also in the discovery cohort was removed from GoSHARE. The same data linkage procedure and phenotype definition criteria for the discovery cohort were applied to this dataset.

### Phenotype Definition Criteria

A phenotype of thyroid replacement therapy was defined as having been issued at least two prescriptions of L-thyroxine (British National Formulary codes-BNF 6.2.1) during the study period. Patients with any prescription of liothyronine and/or with history of thyroid cancer or probable hyperthyroidism were excluded. Unexposed patients never received a prescription for L-thyroxine, and had a serum TSH within the reference range. There was no distinction between AF and atrial flutter when identifying AF phenotypes because the conditions are similar with respect to risk factors and possible complications (Chaker et al., 2015). See Supplementary Material for more detailed information on phenotype definition.

Serum TSH and free thyroxine (FT4) were taken as the median of these measures recorded throughout the study period for each patient.

### Genetic Data

A GWAS-identified *INSR* locus (rs4804416) associated with average serum TSH concentrations and replicated in the GoDARTS cohort (Soto-Pedre et al., 2017) was the candidate gene. To strengthen the choice of this candidate, additional single-nucleotide polymorphisms (SNPs) associated with TSH were also considered regarding AF in patients on L-thyroxine (see Supplementary Table 1). Genotype data was available from several platforms as previously described (Soto-Pedre et al., 2017; see Supplementary Material).

As serum TSH has been identified as a possible underlying mechanism for AF (Ellervik et al., 2019; Salem et al., 2019), a genetic risk score was developed using a weighted sum of TSH increasing alleles (wGRS) reported by Teumer et al. (2018). The purpose of this was to explore whether any possible association with the insulin receptor was driven by serum TSH.

### Statistical Analysis

ANOVA and chi-square tests were used to compare means and frequencies among subgroups of patients, respectively, and non-parametric tests were used where appropriate. Single-locus tests of association with rs4804416 (*INSR*) were performed under the assumption of an additive model in the discovery cohort, with the T/T genotype (ancestral allele) as the reference. Cross-sectional analysis was done by logistic regression models to strengthen the choice of the candidate gene.

Longitudinal analyses to explore the relationship between exposure to L-thyroxine and AF by genotype over time were performed by Cox proportional hazards regression models. The effects of the exposure were estimated by including in models gender, age, and body mass index (BMI) at baseline, and terms for average serum TSH (or FT4) and a diagnosis of diabetes mellitus at any time over the study period were considered as strata. A two-way interaction term between L-thyroxine and genotype was included in all models. To further explore the hypothesis that serum TSH might be related with the development of AF in the context of L-thyroxine therapy, Cox survival models were fitted instead with a two-way interaction term between L-thyroxine and the generated TSH-wGRS (Teumer et al., 2018). The estimates of interaction effects are based on a ratio of hazard ratios (RHR); for each Cox model, ratios between treated (i.e., L-thyroxine) and untreated hazard ratios (HR) were computed. Sensitivity analyses to assess the robustness of the findings were conducted by competing risk regression models where death was the competing event. Model specification was evaluated using goodness of fit diagnostics by computing Harrell’s *C* coefficient, and a test of the proportional hazards assumption was performed for each covariate and globally using a formal significance test based on Schoenfeld residuals. Replication analyses in GoSHARE consisted of repeating same statistical models that were fitted in GoDARTS. Data were entered into a STATA/SE^®^ version 13.1 package (StataCorp, TX, United States) for statistical analysis.

**FIGURE 1:**
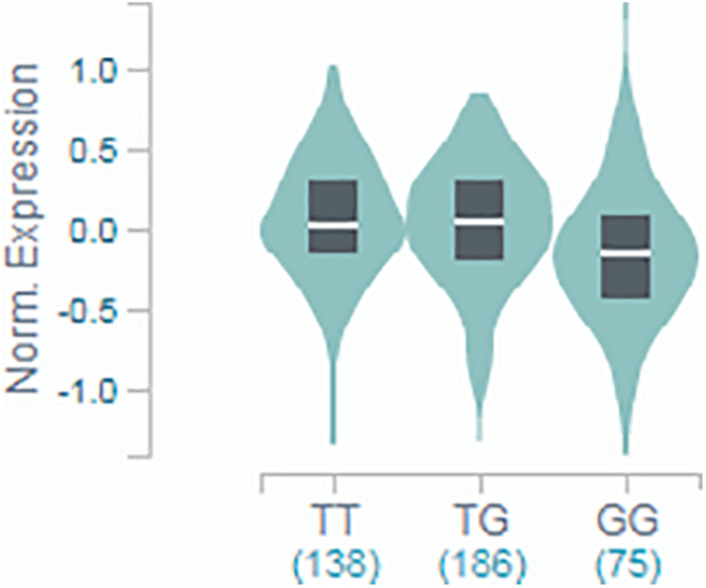
GTEx portal data showing the association of rs4804416 on *INSR* expression in thyroid tissue. Normalized expression in tissue with T/T genotype was 0.017, in T/G was 0.036, and G/G was –0.146 (*T*-test statistic = –3.0, *P* = 0.0034).

The results from the discovery and replication cohorts were combined in a fixed-effect meta-analysis by using R-package “metaphor”^2^, and heterogeneity was quantified using the I-squared measure. Meta-analyses were based on the assumption of an underlying recessive genetic model. Evidence for this effect came from our primary analyses (see Supplementary Tables 2–4), where the effect of the genotype on AF was visibly similar in participants homozygous for the ancestral allele (T/T) and heterozygous carriers (T/G), while the only clear effect was observed in homozygous carriers of the rare allele (G/G). This was supplemented by mRNA expression data available from the Genotype-Tissue Expression (GTEx) portal, where the association of the genotype with *INSR* expression in the thyroid tissue showed a decreased expression only for those with the G/G genotype, while the other two groups showed very marginal increase (see Figure 1).

## Results

We identified 6,802 patients eligible for the study cohort who were *INSR*-rs4804416 genotyped. During a median follow-up of 12.1 years (interquartile range 7.9–15.8 years) a total of 535 AF events occurred. A comparison of the baseline characteristics of the exposed and unexposed cohorts is shown in Table 1. Although those exposed to L-thyroxine had a higher average serum TSH (2.7 vs 1.8 mIU/L, *P* < 1e-03), average TSH (and FT4) levels were within the biochemical reference range. Preliminary cross-sectional analyses strengthen the choice of the candidate gene by showing association signal only for rs4804416 among other SNPs related also to average serum TSH concentration (see Supplementary Table 1).

**TABLE 1:** Description of patients on thyroid replacement therapy (L-thyroxine) and their comparison cohort at study entry (*n* = 6,802).

Characteristic	L-THYROXINE (N = 962)	Comparison cohort (*n* = 5,840)	*P*
**n (%)**			
Gender-female	644 (66.9)	2,205 (37.7)	<0.001
SIMD quintile:			
1 Most deprived	180 (18.9)	1,048 (18.2)	=0.968
2	157 (16.5)	958 (16.7)	
3	155 (16.3)	958 (16.7)	
4	294 (30.9)	1,755 (30.5)	
5 Most affluent	164 (17.2)	1,031 (17.9)	
Diabetes mellitus	236 (24.5)	584 (10.0)	<0.001
Genotype rs4804416:			
TT	323 (33.6)	1,955 (33.5)	=0.962
TG	466 (48.4)	2,813 (48.1)	
GG	173 (18.0)	1,072 (18.4)	
**Mean (SD)**			
Age-years	58.1 (12.5)	59.6 (12.4)	<0.001
BMI (Kg/m^2^)	31.1 (6.6)	30.6 (5.6)	<0.001
Height (cm)	163.9 (9.3)	168.3 (9.6)	<0.001
Serum TSH (mIU/L)*	2.7 (1.7–3.6)	1.8 (1.3–2.4)	<0.001
Serum FT4 (pmol/L)*	14.6 (13.2–16.3)	14.7 (13.3–16.5)	=0.415

BMI, Body mass index; FT4, free thyroxine; SIMD, Scottish Index of Multiple Deprivation; and TSH, Thyroid-stimulating hormone.

* median (interquartile range) of measures recorded throughout the study period.

**FIGURE 2:**
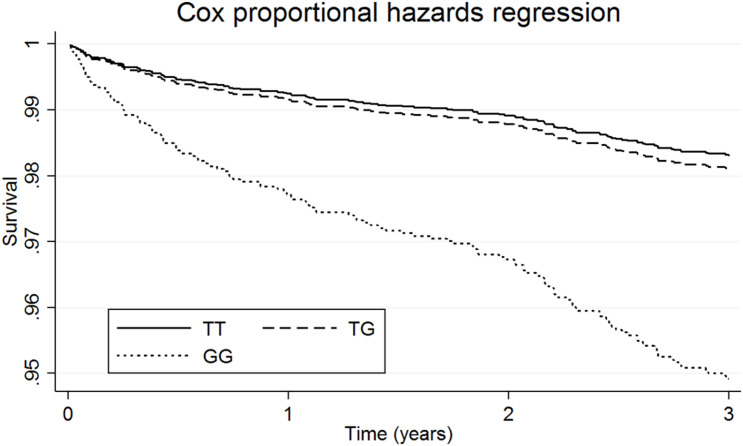
Survival functions of atrial fibrillation in patients on L-thyroxine by genetic variation at *INSR*- rs4804416 within 3 years of follow-up.

Survival analyses showed that for patients taking L-thyroxine there was a significant increased risk of AF for homozygous carriers of the G allele at any time during the follow-up compared to the other genotypes. Table 2 shows the unadjusted and adjusted RHR for the interaction effects between L-thyroxine treatment and genotype on AF risk by time to follow-up. The increased risk was highest within the first 3 years after starting on treatment when it was over nine times higher than in non-carriers (RHR = 9.10, *P* = 8.5e–04). However, heterozygous carriers did not show a significant increased risk compared to non-carriers. Similar results were obtained after adjusting for height instead of BMI or stratifying for serum FT4 instead of TSH (see Supplementary Tables 2, 3, respectively). Figure 2 graphically shows the difference in AF-free survival by genotype within 3 years of treatment. Survival models fitted instead with a two-way interaction term between L-thyroxine and the increasing TSH-wGRS (i.e., weighted TSH-based genetic risk score) showed no associated risk of AF per genetically predicted increase of TSH levels for those treated (see Supplementary Table 5).

**TABLE 2:** Pharmacogenetics interaction between exposure to L-thyroxine and INSR-rs4804416 on developing atrial fibrillation by follow-up time (*n* = 6,802).

Follow-up	At risk (p-y)	Events (*n*)	Genotype	RHR (95% CI)^a^	*P*^†^	RHR (95% CI)^b^	*P*^†^	RHR (95% CI)^c^	*P*^†^
3 years	19,652	128	TG	1.13 (0.38–3.38)	8.2e-01	1.17 (0.39–3.51)	7.7e-01	1.19 (0.39–3.58)	7.5e-01
			GG	7.34 (2.11–25.53)	1.7e-03*	7.48 (2.15–26.06)	1.6e-03*	9.10 (2.48–33.33)	8.5e-04*
5 years	31,837	184	TG	1.04 (0.41–2.60)	9.4e-01	1.06 (0.42–2.68)	8.9e-01	1.10 (0.43–2.78)	8.3e-01
			GG	4.40 (1.62–11.94)	3.6e-03*	4.48 (1.65–12.17)	3.2e-03*	4.70 (1.70–12.95)	2.7e-03*
10 years	57,984	347	TG	1.45 (0.74–2.82)	2.7e-01	1.43 (0.73–2.80)	2.9e-01	1.40 (0.71–2.74)	3.2e-01
			GG	2.77 (1.24–6.19)	1.3e-02*	2.83 (1.26–6.32)	1.1e-02*	2.93 (1.30–6.58)	9.1e-03*
15 years	74,153	470	TG	1.37 (0.75–2.48)	3.0e-01	1.29 (0.71–2.35)	4.0e-01	1.30 (0.71–2.37)	3.9e-01
			GG	2.39 (1.17–4.86)	1.6e-02*	2.36 (1.16–4.81)	1.8e-02*	2.45 (1.20–5.01)	1.4e-02*
20 years	79,301	535	TG	1.49 (0.84–2.65)	1.7e-01	1.45 (0.81–2.57)	2.0e-01	1.45 (0.81–2.58)	2.1e-01
			GG	2.25 (1.12–4.50)	2.2e-02*	2.27 (1.13–4.55)	2.0e-02*	2.35 (1.17–4.72)	1.6e-02*

INSR = insulin receptor-polymorphism. INSR effect allele = G; coding TT = 0, TG = 1, GG = 2. RHR = Ratio of hazard ratios. TSH = thyroid-stimulating hormone.

*P < 0.05, ^†^*P* value for the interaction term (L-thyroxine*INSR).

*^*a*^Unadjusted Cox survival models.*

*^*b*^Adjusted Cox models for age and gender, and stratified by average serum TSH during follow-up and history of diabetes mellitus.*

*^*c*^Adjusted Cox models for age, gender and BMI, and stratified by average serum TSH during follow-up and history of diabetes mellitus.*

Sensitivity analyses using competing risk regression models with death as a competing event are shown in Supplementary Table 4 and yielded similar results to the survival models showed in Table 2. A competing risk is an event that either hinders the observation of the outcome of interest or modifies the chance that this outcome occurs. Thus, our results were not confounded by death.

The replication study consisted of additional 3,190 eligible individuals of white ethnicity recruited from GoSHARE from 1995 to 2018. During a median follow-up of 10.2 years (interquartile range 6.1–14.5 years) a total of 220 AF events occurred. A comparison of the baseline characteristics of the exposed and unexposed groups to L-thyroxine is shown in Supplementary Table 6. Survival analyses showed that for patients taking L-thyroxine there was a significant increased risk of AF for homozygous carriers of the G allele at any time during the follow-up compared to the other genotypes (see Supplementary Figure 1) similar to the discovery cohort. Sensitivity analyses using competing risk regression models with death as a competing event yielded similar results. Supplementary Table 7 shows the increased AF risk in unadjusted and adjusted genetic recessive models, although it was not significant due to less number of AF events in this smaller dataset.

**FIGURE 3:**
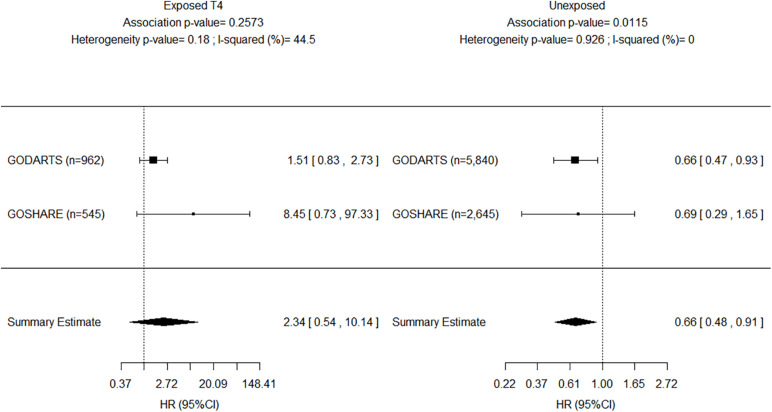
Forest plot for meta-analyses of *INSR*-rs4804416 on developing atrial fibrillation by exposure to L-thyroxine within 10 years of follow-up. HR, Hazard ratio.

**FIGURE 4:**
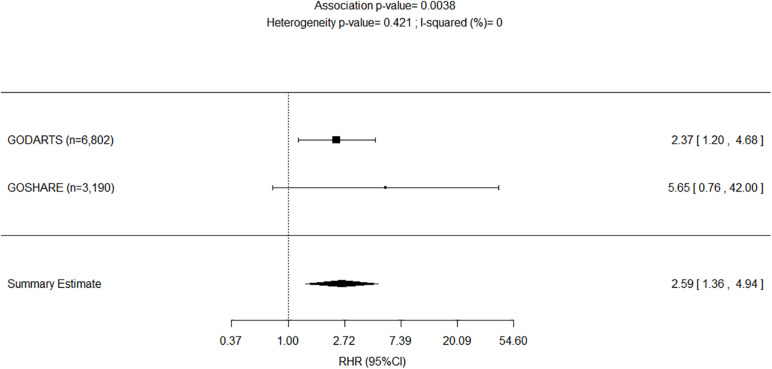
Forest plot for meta-analyses of pharmacogenetics interaction between exposure to L-thyroxine and *INSR*-rs4804416 on developing atrial fibrillation within 10 years of follow-up. RHR, Ratio of hazard ratios.

The results of the adjusted genetic recessive models from the study (i.e., GoDARTS) and replication (i.e., GoSHARE) were further combined in fixed-effect meta-analyses. We reported the *p*-values for the two-tailed test on the combined effect. Figure 3 shows a decreased AF risk for homozygous carriers of rs4804416 G allele in both unexposed cohorts with a significant summary estimate (HR = 0.66, *P* = 1.1e–02), and an increased risk for the exposed cohorts that was not significant due to the sample size (HR = 2.34, *P* = 2.5e–01). To overcome the sample size issue in the exposed groups, a two-way interaction term between L-thyroxine and genotype was included in the model as mentioned earlier. Figure 4 shows a significant summary estimate of interaction effects across the studies (*P* = 3e–03), meaning that homozygous carriers of G allele exposed to L-thyroxine have an increased AF risk 2.59 (95% CI 1.36–4.94) times higher than unexposed individuals. The consistency of the SNP effects direction across the studies (i.e., study and replication) was also graphically shown in Figures 3, 4. Although no significant heterogeneity was detected, a value close to 44% was found in the meta-analysis across the exposed cohorts (*P* = 1.8e–01).

## Discussion

We have undertaken a large follow-up study to assess the association between thyroid hormone replacement therapy, the SNP rs4804416 and risk of developing AF over a 20 year period. We have shown that AF risk is up to nine times higher in patients taking L-thyroxine that are also homozygous carriers of the minor G allele of rs4804416. The impact of L-thyroxine upon AF was evident independent of average serum TSH (or FT4) concentration, BMI (or height) and diabetes status at any point during the follow-up period, which have been associated with AF (Flynn et al., 2010; Rosenberg et al., 2012; Chaker et al., 2015; Baumgartner et al., 2017; Bell and Goncalves, 2019).

Replication was performed in an additional comparable sample (i.e., GoSHARE) where identical phenotype definition criteria were applied. The replication sample was smaller and younger, and thus had fewer AF events during similar average follow-up. Nonetheless, replication results showed same direction of effects, similar effect size when there was no exposure to L-thyroxine, but larger effect size when there was exposure to L-thyroxine. Survival analyses of the discovery and replication data showed similar functions for non-carriers and heterozygous carriers compared to homozygous carriers. Meta-analyses of the association across the discovery and replication were based on underlying recessive genetic models, which did not assume that the underlying genetic model was known in advance but made use of the information available on all genotypes. The association test results observed indicate that variability between estimated effects from the study and the replication can be explained by chance only.

Recently, Salem et al. (2019) and Ellervik et al. (2019) researched the association between thyroid function and AF using genetic data, and provided support for the observational association between thyroid function and AF. They showed that the risk of AF seems to vary throughout the spectrum of thyroid function as measured by TSH, including the reference range. Thus, serum TSH concentration may explain only some of the risk of AF. Salem et al. reported a negative association with AF for carriers of the increasing TSH rs4804416 G allele in a phenome-wide association study of individuals unexposed to L-thyroxine (β = –0.0185, *P* = 0.15; Salem et al., 2019). Ellervik et al. (2019) conducted a Mendelian randomization analysis and reported a protective association of their genetic increasing TSH predictor on AF (OR = 0.88; 95% CI 0.84–0.92). These studies support our finding of decreased AF risk among homozygous carriers of rs4804416 G allele unexposed to L-thyroxine (HR = 0.66; 95% CI 0.48–0.91). Our data provides evidence for the first time that a genetic variation impacts on AF risk in patients treated with L-thyroxine for hypothyroidism. Genetic polymorphisms may increase susceptibility to environmental changes in the pathogenesis of AF (Fatkin et al., 2017). Exposure to L-thyroxine could reverse the protective association of rs4804416 G allele observed in individuals with normal thyroid function.

Candidate genes from GWAS are considered an unbiased approach to identifying or validating genetic influences on drug response (Roden et al., 2011). This approach focuses on associations between genetic variation within pre-specified genes of interest and phenotypes. In this study, a GWAS-identified *INSR* locus (rs4804416) replicated in a Scottish population was the candidate gene (Soto-Pedre et al., 2017). This gene encodes a preproprotein that is processed to generate two alpha and two beta subunits that work together as a functioning insulin receptor. *INSR* gene mutations also underlie the type A insulin resistance syndrome and leads to diabetes mellitus (Semple et al., 2011). Although this SNP has been associated with average serum TSH concentrations, our results are suggestive that the observed increased AF risk for homozygous carriers of the increasing TSH allele (i.e., G/G) in the context of L-thyroxine therapy might not be dependent on serum TSH.

The GTEx Project has created a reference resource of gene expression levels from non-diseased tissues (Carithers and Moore, 2015). Studies of tissue-specific gene expression across human tissues provide useful insights into how genes can affect disease. Data from the GTEx shows that the SNP rs4804416 is a strong expression quantitative trait locus for *INSR* mRNA expression, and it is associated with a statistically significant expression at several tissues (see Supplementary Figure 2). In thyroid and aortic artery tissue, carriers of the rare allele (G) have lower expression of *INSR* mRNA. It is possible that exposure to L-thyroxine associated with impaired expression of *INSR* in patients with the G/G genotype in some of these tissues may explain the mechanism of increased AF risk. This indirectly supports the hypothesis that altered insulin resistance due to reduced mRNA product of *INSR* might be associated with the development of AF in the context of L-thyroxine therapy.

Our results were adjusted for known potential confounders and the impact of missing values is considered low. The data on morbidity and AF mainly related to hospital admission data and would have missed out-patient activity, and thus missed some events of AF underestimating overall AF risk. An apparent limitation of the study is that criteria for individual date of entry into the study differed between exposed and unexposed cohorts; the first date of prescription for exposed to L-thyroxine and the date at first serum TSH recording for those unexposed. However, these criteria do not differ much in clinical practice because serum TSH is nearly always measured before starting on L-thyroxine. A major strength of this study is that it is a longitudinal study and that sensitivity analysis using competing risk regression models with death as a competing event for AF confirmed the results across the discovery and replication datasets.

In summary, genetic polymorphisms in the *INSR* gene may affect disease outcomes in patients on L-thyroxine replacement therapy and support to predict those who will have higher risk of AF. The results of the present study may help to customize L-thyroxine prescribing for patients to improve safety. Further studies are needed to reassure our findings.

## Data Availability Statement

The data analyzed in this study is subject to the following licenses/restrictions: This is consented data and due to sensitive nature is store in secure computing environments. Data can be shared based on specific requests but as such is not publicly available. Requests to access these datasets should be directed to Christopher Hall, C.Hall@dundee.ac.uk.

## Ethics Statement

All analyses were performed on anonymized datasets. The studies involving human participants were reviewed and approved by Tayside Medical Ethics Committee (Scotland, United Kingdom). The patients/participants provided their written informed consent to participate in this study.

## Author Contributions

ES-P researched/analyzed data and wrote the manuscript. MS researched data and wrote the manuscript. CM and AYD researched data and reviewed the manuscript. ASD, CP, and EP contributed to the discussion and reviewed/edited the manuscript. GL planned the study, researched the data, contributed to the discussion, and reviewed/edited the manuscript. All authors contributed to the article and approved the submitted version.

## Conflict of Interest

The authors declare that the research was conducted in the absence of any commercial or financial relationships that could be construed as a potential conflict of interest.

## Footnotes

1 http://www.dundee.ac.uk/hic

2 https://www.r-project.org
