# Supplementary material for: Polymorphism in INSR Locus Modifies Risk of Atrial Fibrillation in Patients on Thyroid Hormone Replacement Therapy
Source: Front Genet. 2021 Jun 23;12:652878. doi: 10.3389/fgene.2021.652878 (PMC8260687; doi:10.3389/fgene.2021.652878)
Supplement: Supplementary file 1 [file Data_Sheet_1.docx]

**Polymorphism in INSR locus modifies risk of atrial fibrillation in patients on thyroid hormone replacement therapy**

Soto-Pedre et al

SUPPLEMENTARY MATERIALS

GENOTYPE DATA

Genotype data was available from several platforms: the Human Exome -12 VI_A_chip, the Metabochip , Illumina HumanOmni Express -12VI platform (Illumina, San Diego), Affymetrix 6.0 platform (Affymetrix, Santa Clara) and the Illumina Infinium custom GWAS chip. Samples with calls rates below 90% had been discarded. Standard quality control measures were performed to exclude single-nucleotide polymorphisms (SNPs) of poor quality including Hardy-Weinberg equilibrium P value <1.0e-04. Imputation had been performed against 1000G Phase I V3 reference panel using Impute2 and using the haplotype reference consortium.

DATA LINKED and PHENOTYPE IDENTIFICATION

Records on redeemed drug prescriptions, demography and biochemistry, the Scottish Care Information-Diabetes Collaboration (SCI-DC) and the Scottish Morbidity Records- SMR (hospital admissions and cancer registry) databases, Office of Population Censuses and Surveys Classification of Surgical Operations version 4 records- OPCS4 (operations, procedures and interventions), and the General Registrar Office-GRO records on patient deaths were linked to genetic data. To ensure data quality, SMR data are routinely subjected to a set of validation rules by the Information Services Division (ISD- NHS National Health Services Scotland), that check on the validity and feasibility of the data (ISD Scotland. *Data quality assurance; assessments of SMR*. <http://www.isdscotland.org/Products-and-Services/Data-Quality/Assessments/>)

Probable hyperthyroidism was considered as having at least one OPCS4 code of treatment with thyroid surgery (OPCS4: B08, B09, and B12), radioactive iodine and/or a prescription of anti-thyroid drug use (BNF 6.2.2). Thyroid cancer was identified from hospital admission International Classification of Diseases (ICD) 9th-10th edition codes (ICD9: 193; ICD10: C73, D093, and D440). AF was identified from ICD codes (ICD9: 427.31, and 427.32; ICD10: I48) resulting in hospital admission.

SUPPLEMENTARY TABLES

Supp.Table 1- Cross-sectional analyses. Genetic association with atrial fibrillation (AF) in patients exposed to L-thyroxine. Single-nucleotide polymorphisms associated with average TSH levels replicated by Soto-Pedre et al. (Pharmacogenet Genomics. 2017; 27: 356-62)

Suppl.Table 2- Longitudinal analyses adjusted for height.

Suppl.Table 3- Longitudinal analyses stratified by serum free thyroxine.

Suppl.Table 4- Longitudinal analyses using competing risk regression models with death as competing event.

Suppl.Table 5- Longitudinal analyses of pharmacogenetics interaction between L-thyroxine and weighted TSH-based genetic risk score (wGRS) on developing AF.

Suppl.Table 6- Replication cohort (GoSHARE). Description of patients exposed to L-thyroxine and their comparison unexposed group at study entry.

Suppl.Table 7- Longitudinal analyses using genetic recessive models by follow-up time and cohort (GoDARTS and GoSHARE)

SUPPLEMENTARY FIGURES

Suppl.Figure 1-Replication cohort (GoSHARE). Survival functions of AF in patients on L-thyroxine by genetic variation at rs4804416 (INSR) within 20 years of follow-up

Suppl.Figure 2-Genotype-Tissue Expression (GTEx) Project. Multi-tissue eQTL Comparison for SNP rs4804416 (INSR)

**Suppl.Table 1** Genetic association with atrial fibrillation in patients on L-thyroxine. Single-nucleotide polymorphisms associated with average TSH levels replicated by Soto-Pedre et al. (Pharmacogenet Genomics. 2017; 27: 356-362)

**SNP CHR Position Coded allele MAF Gene N^†^ OR P**

rs6885099 5 76530349 G 0.39 *PDE8B* 1,752 1.059 5.9e-01

rs10799824 1 19841174 A 0.15 *CAPZB* 1,375 1.025 8.8e-01

rs753760 6 166046483 G 0.30 *PDE10A* 1,374 1.018 8.9e-01

rs3813582 16 79749353 C 0.30 LOC105371356 1,372 1.112 3.9e-01

rs10032216 4 149669506 C 0.21 *NR3C2* 1,374 1.050 7.4e-01

rs9472138 6 43811762 T 0.30 *VEGFA* 1,780 1.024 8.4e-01

rs13015993 2 217625523 A 0.27 *IGFBP5* 1,375 1.007 9.5e-01

rs4804416 19 7223848 G 0.42 *INSR* 1,374 1.236 7.3e-02 *

rs17723470 11 45227567 T 0.30 *PRDM11* 1,375 1.063 6.3e-01

rs334699 1 61620496 G 0.05 *NFIA* 1,373 1.307 3.8e-01

rs11624776 14 93595591 C 0.32 *ITPK1* 1,374 1.014 9.1e-01

rs657152 9 136139265 A 0.31 *ABO* 1,782 0.954 6.7e-01

CHR= chromosome. MAF= Minor allele frequency. TSH= thyroid stimulating hormone. Unadjusted logistic regression models (OR=odds ratio) (†) Number of subjects genotyped (*) P<1e-01

**Suppl. Table 2** Survival models adjusted for height. Pharmacogenetics interaction between exposure to L-thyroxine and INSR-rs4804416 on developing atrial fibrillation by follow-up time (n=6,802).

Follow-up At risk (p-y) Events (n) Genotype RHR (95% CI) ^a^ P† RHR (95% CI) ^b^ P† RHR (95% CI) ^c^ P†

**3 YEARS** 19,652 128 TG 1.13 (0.38- 3.38) 8.2e-01 1.17 (0.39- 3.51) 7.7e-01 1.16 (0.38- 3.50) 7.8e-01

GG 7.34 (2.11- 25.53) 1.7e-03 * 7.48 (2.15- 26.06) 1.6e-03 * 8.84 (2.41- 32.39) 9.9e-04 *

**5 YEARS** 31,837 184 TG 1.04 (0.41- 2.60) 9.4e-01 1.06 (0.42- 2.68) 8.9e-01 1.08 (0.43- 2.74) 8.6e-01

GG 4.40 (1.62- 11.94) 3.6e-03 * 4.48 (1.65- 12.17) 3.2e-03 * 4.65 (1.69- 12.81) 2.9e-03 *

**10 YEARS** 57,984 347 TG 1.45 (0.74- 2.82) 2.7e-01 1.43 (0.73- 2.80) 2.9e-01 1.36 (0.69- 2.68) 3.6e-01

GG 2.77 (1.24- 6.19) 1.3e-02 * 2.83 (1.26- 6.32) 1.1e-02 * 2.87 (1.27- 6.44) 1.1e-02 *

**15 YEARS** 74,153 470 TG 1.37 (0.75- 2.48) 3.0e-01 1.29 (0.71- 2.35) 4.0e-01 1.25 (0.68- 2.29) 4.6e-01

GG 2.39 (1.17- 4.86) 1.6e-02 * 2.36 (1.16- 4.81) 1.8e-02 * 2.38 (1.16- 4.86) 1.7e-02 *

**20 YEARS** 79,301 535 TG 1.49 (0.84- 2.65) 1.7e-01 1.45 (0.81- 2.57) 2.0e-01 1.39 (0.78- 2.48) 2.6e-01

GG 2.25 (1.12- 4.50) 2.2e-02 * 2.27 (1.13- 4.55) 2.0e-02 * 2.28 (1.14- 44.58) 2.0e-02 *

INSR= insulin receptor-polymorphism. INSR effect allele=G; coding TT=0, TG=1, GG=2. RHR=Ratio of hazard ratios. TSH= thyroid-stimulating hormone.

(*) P<5e-02 (†) P value for the interaction term (L-thyroxine*INSR)

a) Unadjusted Cox survival models.

b) Adjusted Cox models for age and gender, and stratified by average serum TSH during follow-up and history of diabetes mellitus.

c) Adjusted Cox models for age, gender and height, and stratified by average serum TSH during follow-up and history of diabetes mellitus.

**Suppl. Table 3** Survival models stratified by serum free thyroxine. Pharmacogenetics interaction between exposure to L-thyroxine and INSR-rs4804416 on developing atrial fibrillation by follow-up time (n=2,380).

Follow-up At risk (p-y) Events (n) Genotype RHR (95% CI) ^a^ P† RHR (95% CI) ^b^ P† RHR (95% CI) ^c^ P†

**3 years**  19,621 128 TG 1.13 (0.38- 3.38) 8.2e-01 1.11 (0.32- 3.84) 8.7e-01 1.05 (0.29- 3.70) 9.4e-01

GG 7.34 (2.11- 25.53) 1.7e-03 * 7.02 (1.37- 36.04) 1.9e-02 * 6.20 (1.19- 32.15) 3.0e-02 *

**5 years**  31,789 184 TG 1.04 (0.41- 2.60) 9.4e-01 1.01 (0.36- 2.83) 9.8e-01 0.98 (0.35- 2.75) 9.7e-01

GG 4.40 (1.62- 11.94) 3.6e-03 * 5.01 (1.42- 17.72) 1.2e-02 * 4.52 (1.27- 16.07) 2.0e-02 *

**10 years**  57,904 347 TG 1.45 (0.74- 2.82) 2.7e-01 1.48 (0.70- 3.13) 3.0e-01 1.39 (0.65- 2.96) 3.9e-01

GG 2.77 (1.24- 6.19) 1.3e-02 * 3.77 (1.39- 10.18) 8.7e-03 * 3.67 (1.35- 9.92) 1.0e-02 *

**15 years**  74,056 470 TG 1.37 (0.75- 2.48) 3.0e-01 1.31 (0.67- 2.55) 4.1e-01 1.24 (0.64- 2.42) 5.1e-01

GG 2.39 (1.17- 4.86) 1.6e-02 * 2.37 (1.03- 5.42) 4.0e-02 * 2.31(1.01- 5.30) 4.7e-02 *

**20 years**  79,200 535 TG 1.49 (0.84- 2.65) 1.7e-01 1.43 (0.76- 2.70) 2.6e-01 1.37 (0.72- 2.59) 3.3e-01

GG 2.25 (1.12- 4.50) 2.2e-02 * 2.22 (1.01- 4.95) 4.9e-02 * 2.17 (0.97- 4.85) 5.7e-02

FT4=free thyroxine. INSR= insulin receptor-polymorphism. INSR effect allele=G; coding TT=0, TG=1, GG=2. RHR=Ratio of hazard ratios

(*) P<5e-02 (†) P value for the interaction term (L-thyroxine*INSR)

a) Unadjusted Cox survival models.

b) Adjusted Cox models for age and gender, and stratified by average serum FT4 during follow-up and history of diabetes mellitus.

c) Adjusted Cox models for age, gender and BMI, and stratified by average serum FT4 during follow-up and history of diabetes mellitus.

**Suppl. Table 4** Competing risk regression models. Pharmacogenetics interaction between exposure to L-thyroxine and INSR-rs4804416 on developing atrial fibrillation by follow-up time (n=6,802).

Follow-up At risk (p-y) Events (n) Genotype RHR (95% CI) ^a^ P† RHR (95% CI) ^b^ P† RHR (95% CI) ^c^ P†

**3 years**  19,652 128 TG 1.14 (0.38- 3.39) 8.1e-01 1.18 (0.40- 3.53) 7.6e-01 1.22 (0.40- 3.68) 7.2e-01

GG 7.35 (2.12- 25.45) 1.6e-03 * 7.65 (2.20- 26.62) 1.4e-03 * 9.44 (2.57- 34.74) 7.2e-04 *

**5 years**  31,837 184 TG 1.04 (0.41- 2.61) 9.3e-01 1.08 (0.43- 2.69) 8.7e-01 1.13 (0.44- 2.84) 7.9e-01

GG 4.38 (1.62- 11.86) 3.6e-03 * 4.53 (1.68- 12.20) 2.8e-03 * 4.85 (1.76- 13.31) 2.2e-03 *

**10 years**  57,984 347 TG 1.47 (0.76- 2.87) 2.5e-01 1.51 (0.77- 2.94) 2.2e-01 1.49 (0.75- 2.92) 2.5e-01

GG 2.76 (1.23- 6.19) 1.4e-02 * 2.87 (1.27- 6.48) 1.1e-02 * 2.98 (1.31- 6.79) 9.0e-03 *

**15 years**  74,153 470 TG 1.39 (0.77- 2.53) 2.7e-01 1.40 (0.76- 2.56) 2.7e-01 1.40 (0.76- 2.58) 2.7e-01

GG 2.36 (1.16- 4.83) 1.8e-02 * 2.39 (1.16- 4.94) 1.8e-02 * 2.47 (1.19- 5.11) 1.5e-02 *

**20 years**  79,301 535 TG 1.50 (0.85- 2.67) 1.6e-01 1.50 (0.84- 2.69) 1.7e-01 1.50 (0.83- 2.71) 1.7e-01

GG 2.20 (1.09- 4.44) 2.7e-02 * 2.22 (1.09- 4.52) 2.7e-02 * 2.27 (1.11- 4.64) 2.3e-02 *

BMI= Body mass index. INSR= insulin receptor-polymorphism. INSR effect allele=G; coding TT=0, TG=1, GG=2. RHR=Ratio of hazard ratios TSH= thyroid-stimulating hormone.

(*) P<5e-02 (†) P value for the interaction term (L-thyroxine*INSR)

Modified Cox survival models with death as competing event: a) unadjusted models, b) adjusted for age and gender, c) adjusted for age, gender and BMI.

**Suppl. Table 5** Pharmacogenetics interaction between exposure to L-thyroxine and weighted TSH-based genetic risk score (wGRS) on developing atrial fibrillation by follow-up time (n=5,351).

Follow-up At risk (p-y) Events (n) RHR (95% CI) ^a^ P† RHR (95% CI) ^b^ P†

**3 years**  15,480 112 0.87 (0.58- 1.30) 5.1e-01 0.86 (0.57- 1.30) 4.9e-01

**5 years**  25,130 158 0.91 (0.64- 1.29) 6.0e-01 0.89 (0.62- 1.27) 5.3e-01

**10 years**  46,063 296 0.90 (0.69- 1.18) 4.7e-01 0.89 (0.68- 1.17) 4.3e-01

**15 years**  59,177 404 0.89 (0.70- 1.13) 3.4e-01 0.88 (0.69- 1.12) 3.0e-01

**20 years**  63,310 463 0.87 (0.69- 1.09) 2.3e-01 0.86 (0.68- 1.08) 2.2e-01

BMI= Body mass index. RHR=Ratio of hazard ratios. TSH= thyroid-stimulating hormone. wGRS= weighted genetic risk score quartiles (Q1=3.57- 4.75, Q2=4.76- 4.98, Q3=4.99- 5.19, Q4=5.20- 6.16). (†) P value for the interaction term (L-thyroxine*wGRS)

a) Unadjusted Cox survival models

b) Adjusted Cox models for age, gender and BMI, and stratified by history of diabetes mellitus.

**Suppl.Table 6** Replication cohort (GoSHARE). Description of patients on thyroid replacement therapy (L-thyroxine) and their comparison cohort at study entry (n=3,190)

L-thyroxine Comparison cohort

CHARACTERISTIC (n= 545) (n= 2,645) P

***n (%)***

Gender-female 384 (70.59) 1,015 (38.4) <1e-03

SIMD quintile:

1 most deprived 87 (16.5) 388 (15.1) =6.6e-01

2 73 (13.8) 329 (12.8)

3 79 (14.9) 437 (17.0)

4 183 (34.7) 867 (33.7)

5 most affluent 106 (20.1) 547 (21.3)

Diabetes Mellitus 193 (35.4) 75 (2.8) <1e-03

Genotype rs4804416:

TT 158 (29.0) 877 (33.1) =1.6e-01

TG 283 (51.9) 1,294 (48.9)

GG 104 (19.1) 474 (17.9)

***Mean (SD)***

Age-years 53.9 (12.6) 56.6 (12.2) <1e-03

BMI (Kg/m^2^) 31.8 (6.5) 30.3 (5.8) <1e-03

Height (cm) 165.2 (10.6) 168.6 (10.2) <1e-03

Serum TSH (mU/l) * 2.4 (1.5- 3.2) 1.7 (1.2- 2.3) <1e-03

Serum FT4 (pmol/L) * 14.4 (12.7- 16.2) 15.3 (12.7- 18.9) <1e-03

BMI= Body mass index. FT4= free thyroxine. SIMD= Scottish Index of Multiple Deprivation. TSH= Thyroid-stimulating hormone. (*) median (interquartile range) of measures recorded throughout the study period

**Suppl.Table 7** Genetic recessive models. Pharmacogenetics interaction between exposure to L-thyroxine and INSR-rs4804416 on developing atrial fibrillation by cohort and follow-up time.

Follow-up At risk (p-y) Events (n) Genotype RHR (95% CI) ^a^ P† RHR (95% CI) ^b^ P† HR (95% CI) ^c^ P†

**GoDARTS (n=6,802)**

10 years 57,984 347 GG 2.19 (1.12- 4.29) 2.2e-02* 2.25 (1.14- 4.41) 1.8e-02* 2.37 (1.20- 4.67) 1.3e-02*

15 years 74,153 470 GG 1.96 (1.08- 3.57) 2.6e-02* 2.01 (1.10- 3.66) 2.1e-02* 2.09 (1.14- 3.81) 1.6e-02*

20 years 79,301 535 GG 1.75 (1.03- 3.31) 3.8e-02* 1.80 (1.01- 3.24) 4.6e-02* 1.87 (1.04- 3.36) 3.6e-02*

**GoSHARE (n=3,190)**

10 years 27,239 138 GG 2.87 (0.98- 8.38) 5.3e-02 5.76 (0.89- 37.07) 6.5e-02 5.65 (0.76- 41.98) 9.1e-02

15 years 36,078 192 GG 2.24 (0.90- 5.57) 8.0e-02 2.88 (0.57- 14.55) 2.0e-01 2.35 (0.41- 13.39) 3.4e-01

20 years 40,785 220 GG 1.84 (0.79- 4.26) 1.5e-01 1.51 (0.36- 6.33) 5.7e-01 1.15 (0.24- 5.47) 8.6e-01

INSR= insulin receptor-polymorphism. INSR effect allele=G; coding TT/TG=0, GG=1. RHR=Ratio of hazard ratios. TSH= thyroid-stimulating hormone.

(*) P<5e-02 (†) P value for the interaction term (L-thyroxine*INSR)

a) Unadjusted Cox survival models.

b) Adjusted Cox models for age and gender, and stratified by average serum TSH during follow-up and history of diabetes mellitus.

c) Adjusted Cox models for age, gender and BMI, and stratified by average serum TSH during follow-up and history of diabetes mellitus

SUPPLEMENTARY FIGURES

**Suppl.Figure 1** Replication study (GoSHARE). Survival functions of atrial fibrillation in patients on L-thyroxine by genetic variation at rs4804416 (INSR) within 20 years of follow-up.

**Suppl.Figure 2** Genotype-Tissue Expression (GTEx) Project. Multi-tissue eQTL Comparison for SNP rs4804416 (INSR).
