# Supplementary figures and images for: Polymorphism in INSR Locus Modifies Risk of Atrial Fibrillation in Patients on Thyroid Hormone Replacement Therapy
Source: Front Genet. 2021 Jun 23;12:652878. doi: 10.3389/fgene.2021.652878 (PMC8260687; doi:10.3389/fgene.2021.652878)

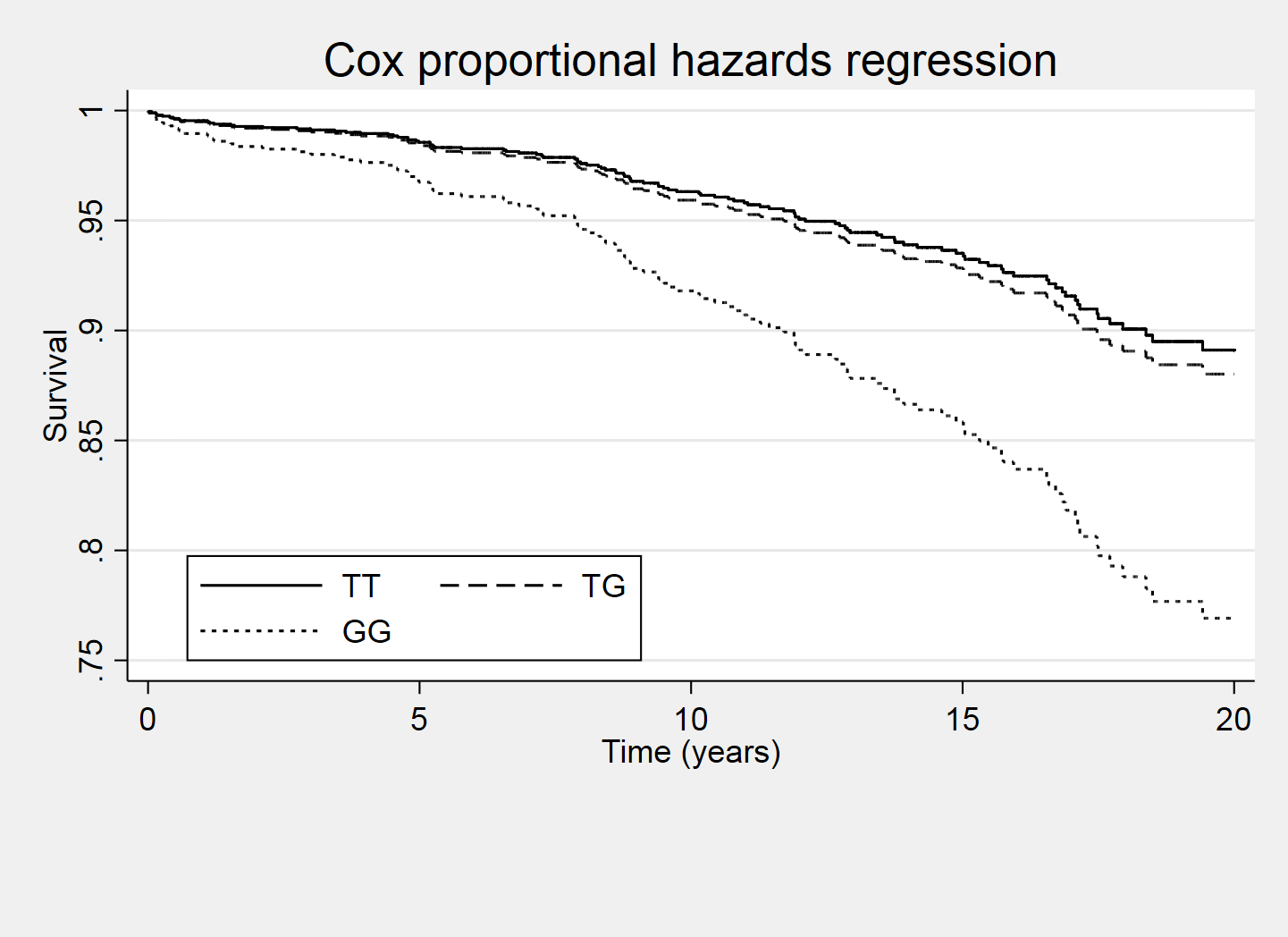

Supplement: Supplementary file 2 [file Image_1.TIF]

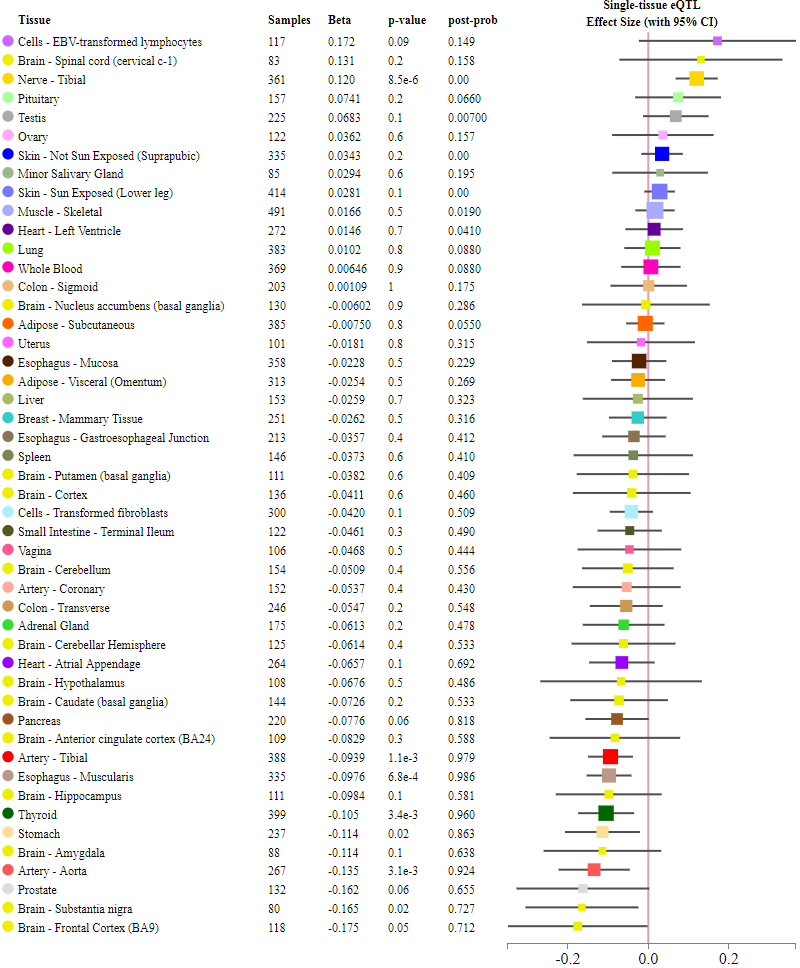

Supplement: Supplementary file 3 [file Image_2.TIF]
